# Comprehensive analysis of GINS subunits prognostic value and ceRNA network in sarcoma

**DOI:** 10.3389/fcell.2022.951363

**Published:** 2022-08-26

**Authors:** Chuqiao Zhou, Zhuoyuan Chen, Bo Xiao, Cheng Xiang, Aoyu Li, Ziyue Zhao, Hui Li

**Affiliations:** ^1^ Department of Orthopedics, The Second Xiangya Hospital, Central South University, Changsha, China; ^2^ Orthopedic Biomedical Materials Engineering Laboratory of Hunan Province, Changsha, China

**Keywords:** sarcoma, GINS, prognosis, immune infiltration, bioinformatics analysis

## Abstract

**Background:** The GINS complex, composed of GINS1/2/3/4 subunits, is an essential structure of Cdc45-MCM-GINS (CMG) helicase and plays a vital role in establishing the DNA replication fork and chromosome replication. Meanwhile, GINS genes have been associated with the poor prognosis of various malignancies. However, the abnormal expression of GINS genes and their diagnostic and prognostic value in sarcomas (SARC) remain unclear.

**Methods:** Oncomine, Gene Expression Profiling Interactive Analysis (GEPIA), Kaplan-Meier Plotter, Cancer cell line encyclopedia (CCLE), The University of Alabama at Birmingham Cancer Data Analysis Portal (UALCAN), R studio, and Tumor Immune Estimation Resource (TIMER) were used to analyze the expression profiles, prognostic value, biological function, ceRNA, and immune infiltration associated with GINS genes in sarcomas.

**Results:** We found that GINS1/2/3/4 genes exhibited significantly upregulated transcription levels in SARC samples compared to non-tumor tissues and exhibited high expression levels in sarcoma cell lines. In addition, SARC patients with increased expression levels of GINS1/2/3/4 showed poorer survival rates. Immune infiltration analysis showed that GINS subunits were closely associated with the infiltration of immune cells in sarcomas.

**Conclusion:** Our research identified GINS subunits as potential diagnostic and prognostic biological targets in SARC and elucidated their underlying effects in the genesis and progression of SARC. These results may provide new opportunities and research directions for targeted sarcoma therapy.

## Introduction

Sarcomas (SARC) are rare and malignant mesenchymal neoplasms, mainly composed of soft tissue and bone sarcomas. They can occur anywhere in the body, but are most common in the extremities, accounting for about 1% of adult malignancies and 20% of solid tumors in children (Bleloch et al., 2017). It is well known that early complete surgical resection is the key to prolonging patients’ survival time with malignant tumors. However, most sarcoma patients do not have typical clinical symptoms early on and are often not diagnosed until the symptoms are evident at an advanced stage when R0 resection is not achievable. The therapeutic landscape for sarcomas has undergone little change over the past decade, while the median overall survival for terminal soft tissue sarcoma patients is only 12–19 months, although systemic therapy and local radiotherapy, ablation, or surgery are available (Younger et al., 2021). As current treatments are not satisfactory for sarcoma patients, early diagnosis and more effective therapies are needed to improve patient outcomes. Due to the heterogeneity of sarcomas and their multiple histological subtypes of different clinical and biological behaviors, it has been challenging to develop new approaches for early diagnosis and personalized treatment.

The GINS complex was first discovered in *Saccharomyces cerevisiae* by Japanese scientists Yuko Takayama et al. (2003). The GINS complex is a tetrameric protein complex encoded by GINS family genes which consist of GINS1/2/3/4. The GINS complex is essential for initiating and continuing eukaryotic chromosome replication by maintaining the stability of replication forks and mediating the interaction of many replication factors, as it is a crucial component of the Cdc45-MCM-GINS (CMG) helicase in eukaryotes (Labib and Gambus, 2007). Previous studies have associated the GINS subunits with the cell cycle, cell proliferation, and cell differentiation. For example, GINS1 was upregulated in immature cells and tissues with high proliferation (Ueno et al., 2005). Meanwhile, GINS1 expression is critical for stabilizing the hematopoietic cell pool size, promoting mesenchymal stem cell-mediated bone marrow regeneration, and maintaining early embryogenesis in mice, while the loss of GINS1 leads to the death of mouse embryos (Yoshida et al., 2017). GINS2 is closely associated with retinal cell differentiation in *Xenopus laevis*, and knockdown of GINS2 promotes retinal and lens development (Walter et al., 2008). Moreover, when GINS2 expression levels are reduced, DNA packaging becomes more compact, affecting transcriptional regulation (Chmielewski et al., 2012). It has been shown that GINS3 gene mutation affects myocardial repolarization and may be associated with abnormal differentiation of cardiac myocytes (Newton-Cheh et al., 2009). GINS4 is essential for cell proliferation in mammals, and its expression is closely related to cell cycle regulation and repair of damaged DNA in normal cells (non-tumor cells), suggesting it is essential for maintaining genomic integrity (Gouge and Christensen, 2010; Gong et al., 2014).

It is well-established that GINS subunits play an essential role in DNA replication, which is closely related to one of the characteristics of oncogenesis and tumor progression, the abnormal cell cycle and proliferation. Therefore, in recent years, the GINS genes have been widely studied as potentially therapeutic and prognostic biomarkers of malignant tumors. An increasing body of evidence from recently published studies suggests that GINS gene upregulation is closely associated with a variety of malignancies, including pancreatic, liver, colorectal, lung, ovarian cancers and gastric adenocarcinoma (Bu et al., 2020; Rong et al., 2020; Feng et al., 2021; Sun et al., 2021; Zhan et al., 2021; Zhang et al., 2021; Hu et al., 2022). Meanwhile, GINS high expression has been associated with tumor metastasis, hormone sensitivity, and invasiveness and affected the survival of patients (Peng et al., 2016). Hence, GINS genes are biological targets of crucial clinical significance. Nonetheless, the effect and regulatory mechanism of GINS subunits in sarcoma progression and their prognostic value in sarcoma remain unclear. Accordingly, using bioinformatics methods to systematically analyze GINS expression and its role in prognosis, immune infiltration, and genetic changes, as well as its possible mechanisms in SARC, will further deepen our understanding of the pathogenesis of SARC and provide a new direction for targeted therapy.

**FIGURE 1:**
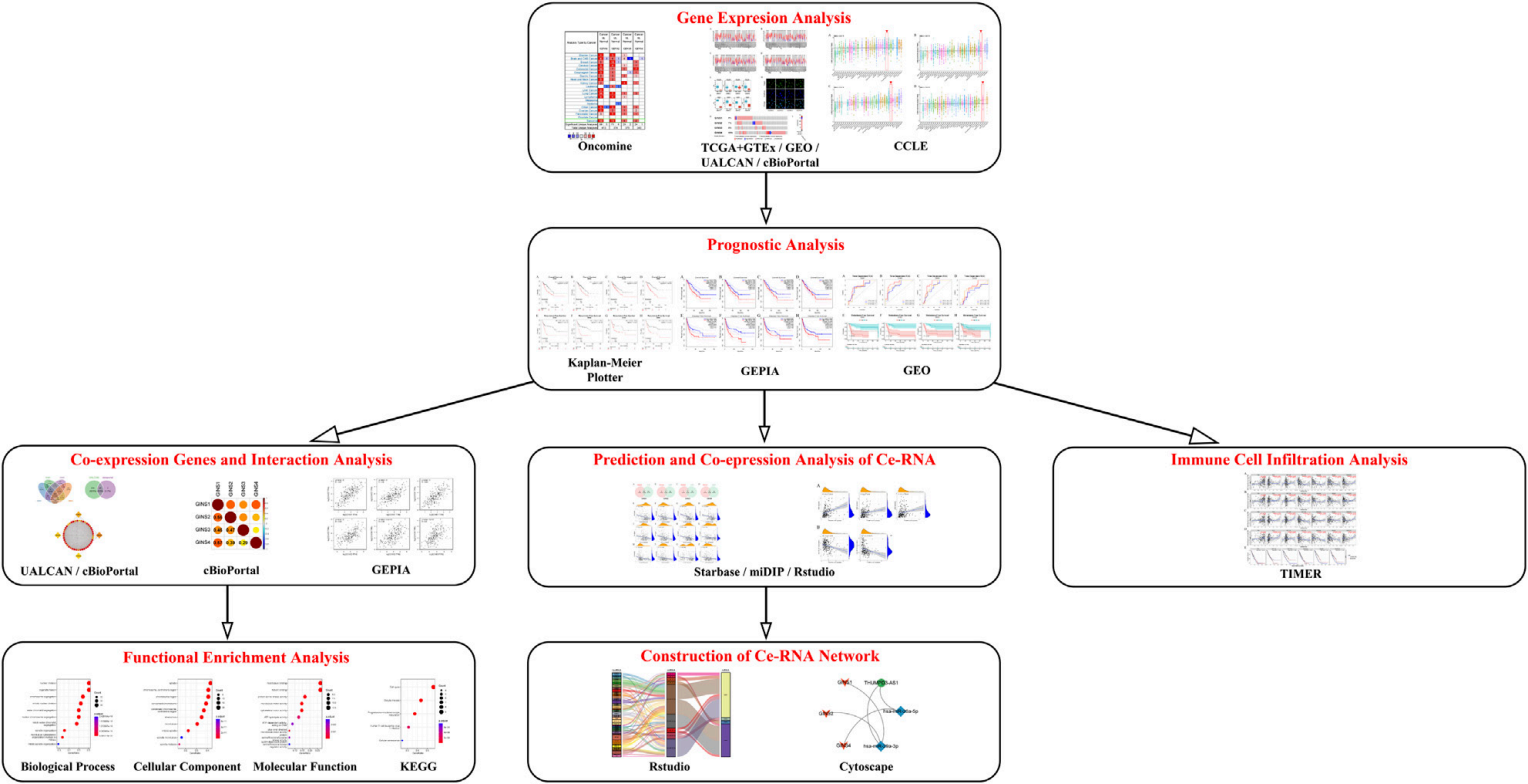
Schematic diagram of this study.

In the present study, various bioinformatics tools, including Oncomine, the Tumor Immune Estimation Resource (TIMER), The Cancer Genome Atlas (TCGA), Genotype-Tissue Expression (GTEx) and Cancer cell line encyclopedia (CCLE) databases, were utilized to detect the GINS expression profiles in SARC. Kaplan-Meier Plotter and Gene Expression Profiling Interactive Analysis (GEPIA) databases were used to evaluate the effect of GINS genes on survival in SARC. The relationship between GINS subunits expression and immune cell infiltration in SARC was explored using the TIMER database. In addition, we identified enriched biological functions, signaling pathways, and the ceRNA network of GINS subunits and their co-expression genes by the Rstudio software. The schematic diagram of this study is illustrated in Figure 1.

## Materials and methods

### Data obtainment

The RNA transcription data and corresponding clinical data of the sarcoma sample were downloaded from the TCGA-SARC dataset. After excluding samples with incomplete RNA transcription data, the dataset contained 259 primary tumors and 2 normal tissue samples from 259 sarcoma cases. The pathological classification of the sarcoma samples included leiomyosarcomas (*n* = 104), dedifferentiated liposarcomas (*n* = 59), undifferentiated sarcomas (*n* = 34), fibromyxosarcomas (*n* = 25), malignant fibrous histiocytomas (*n* = 12), synovial sarcomas (*n* = 10), malignant peripheral nerve sheath tumor (*n* = 9), and other types (*n* = 6). Meanwhile, the RNA transcription data of 515 normal adipose tissue were downloaded from the GTEx database. Furthermore, the transcribed data and correlated information of the two GEO datasets, GSE68591 and GSE40021, were downloaded and analyzed by the R package GEOquery. The GSE68591 dataset included mRNA transcription data of 68 human sarcoma cell lines and 5 normal human cells. The GSE40021 dataset included mRNA transcription data from 53 primary synovial sarcoma samples after excluding samples with no follow-up period.

### Oncomine database analysis

Oncomine (www.oncomine.org) is the largest comprehensive bioinformatics analysis platform comprising 86,733 tumor and non-tumor samples from 715 databases (Pan et al., 2019; Zou et al., 2021). Therefore, we analyzed the expression of GINS subunits in sarcoma and non-tumor samples *via* the Oncomine database. The criteria for filtering datasets included a *p*-value of 0.01, a fold change of 2, and genes ranked in the top 10% (Sun et al., 2019).

### Tumor immune estimation resource dataset analysis

TIMER is a bioinformatics website that comprehensively evaluates RNA expression and multiple immune cell infiltrations in 10,897 samples of 32 cancers from the TCGA database (Li et al., 2017). In this study, the TIMER database was used to verify the significant difference in GINS subunits expression level between tumor and non-tumor tissues. Meanwhile, the TIMER database was used to investigate the relationship between the transcription levels of GINS genes and immune cell infiltration in SARC (Chen et al., 2020).

### Kaplan-Meier plotter database analysis

Kaplan-Meier plotter is an online tool that analyzes the survival effects of 54,675 genes from 10,461 cancer samples across 21 cancer types (Lanczky and Gyorffy, 2021). The Log-rank *p*-value, HRs, 95% CI, and survival curve were calculated and displayed on the web page (Li et al., 2020b). Moreover, we analyzed the effect of the mRNA transcription level of GINS genes on survival in SARC by Kaplan-Meier Plotter.

### Gene expression profiling interactive analysis database analysis

GEPIA is a bioinformatics tool that comprehensively analyzes RNA expression sequencing from 9,736 tumors and 8,587 non-tumor samples, from TCGA and GTEx databases (Tang et al., 2017). The GEPIA database was used for single-gene survival analysis to assess the prognostic value of GINS.

### cBioPortal database analysis

The cBioPortal for Cancer Genomics is an online bioinformatics site for multi-dimensional, visual analysis of cancer genomes from multiple cancer genome databases (Gao et al., 2013). Through the cBioPortal website, we obtained the mutation profiles, mRNA expression levels, and neighbor genes of GINS1/2/3/4 in sarcoma. The threshold value of mRNA expression z scores was set as ± 2.0 (Zhang et al., 2020a).

### Cancer cell line encyclopedia database analysis

CCLE is an online bioinformatics database containing the genetic characteristics of 1072 cell lines for various cancers (Ghandi et al., 2019). This study analyzed the mRNA transcription of GINS genes in different cancer cell lines using CCLE data to further understand GINS genes expression in sarcoma.

### University of alabama at birmingham cancer data analysis portal analysis

The University of Alabama at Birmingham Cancer Data Analysis Portal (UALCAN) is a bioinformatics website for comprehensive analyzing RNA-seq data and clinical information for 31 cancer types in TCGA (Chandrashekar et al., 2017). To identify the co-expressed genes of GINS1/2/3/4 in SARC, we retrieved the co-expressed genes of GINS1/2/3/4 through UALCAN, and then intersected these results.

### Functional and pathway enrichment analyses

The enrichment analysis was performed, and clusters were visualized by the R package software of clusterProfiler (v 4.2.0) (Yu et al., 2012). The present study conducted Gene ontology (GO) and Kyoto Encyclopedia of Genes and Genomes (KEGG) pathway enrichment analysis of GINS subunits and their related genes in RStudio. GO enrichment analysis predicted gene function from biological process (BP), cellular component (CC), and molecular function (MF). A *p*-value < 0.05 was statistically significant (Gong et al., 2021).

### The ceRNA network construction

The starBase and mirDIP websites were used to predict miRNAs targeting GINS1/2/3/4 genes, and lncRNAs binding the target miRNA were predicted by starBase (Li et al., 2014; Tokar et al., 2018). The expression correlations between target miRNA and GINS gene, as well as lncRNA and target miRNA, were verified by co-expression analysis using the R package limma (v 3.50.0) based on the TCGA-SARC dataset. Our co-expression analysis set the threshold value of the *p*-value as < 0.005.

### Statistical analysis

The R (v 4.1.0) and online analysis tools were used to perform all statistical analyses. We used R package ggpubr (v 0.4.0), ggplot2 (v 3.3.5), corrplot (v 0.92), clusterProfiler (v 4.2.0) and limma (v 3.50.0) to visualize data. The Wilcoxon rank-sum test was conducted to analyze the differential expression of GINS genes between sarcoma and non-tumor samples. The log-rank test was performed to compare survival time between high and low gene expression groups of SARC patients. The Spearman test was applied for correlation expression analysis between genes. The threshold of statistically significant was set as *p*-value < 0.05.

## Results

### The expression of GINS subunits in sarcomas patients

**FIGURE 2:**
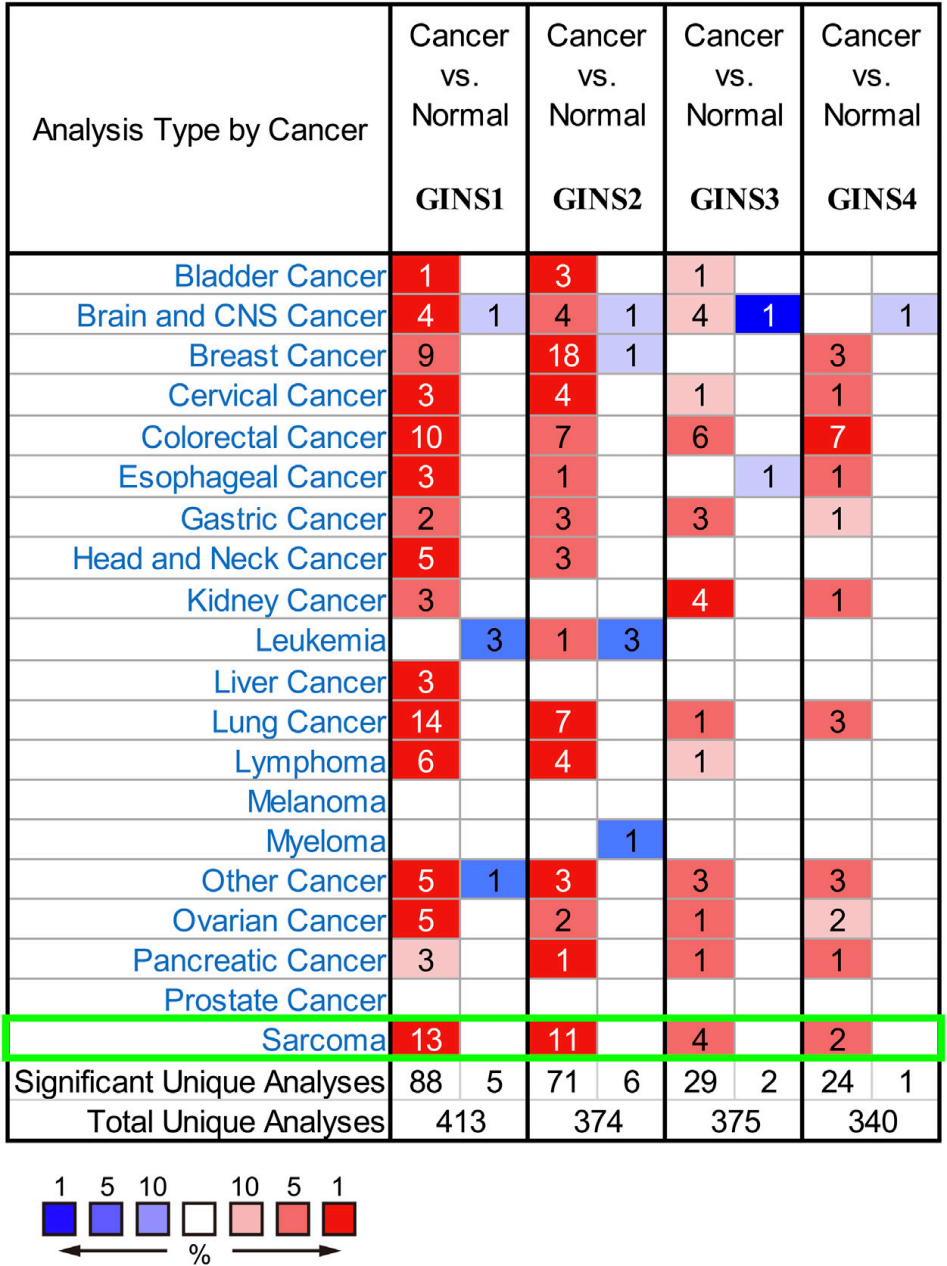
The mRNA transcription of GINS subunits in SARC in Oncomine. Upregulated expression is shown in red and downregulated expression is shown in blue. The values in the cells represent the number of datasets that meet the threshold.

**FIGURE 3:**
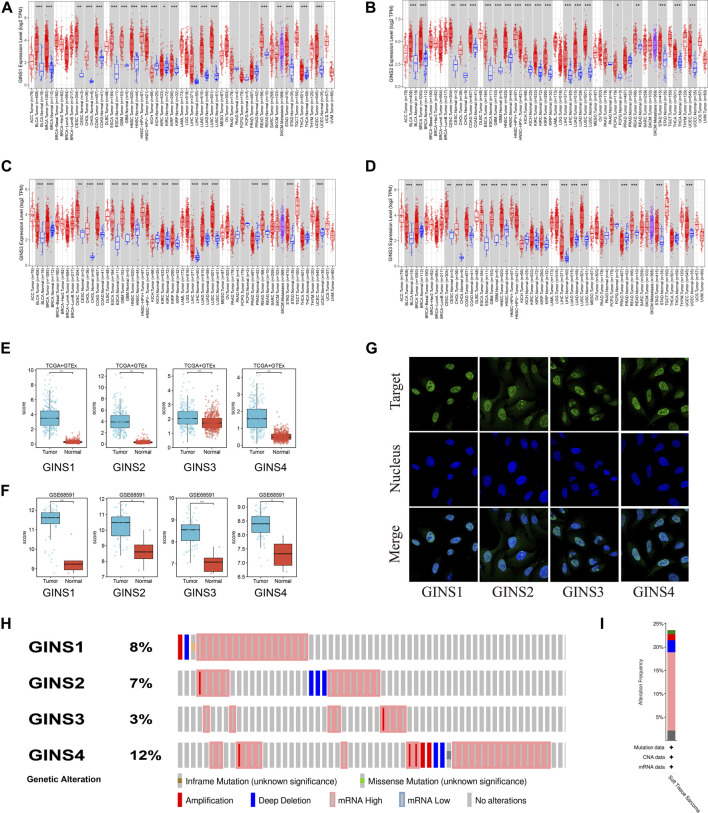
The expression alteration of GINS subunits in sarcoma. Expression levels of GINS1/2/3/4 in pan-cancer **(A–D)**. Expression levels of GINS1/2/3/4 in sarcoma in TCGA and GTEx database **(E)**. Expression levels of GINS1/2/3/4 in sarcoma in GSE68591 dataset **(F)**. The immunofluorescence images exhibited GINS1/2/3/4 proteins (green) and nucleus (blue) co-localization in the osteosarcoma cell line (U2OS) **(G)**. Frequency and type of alteration of GINS1/2/3/4 in sarcoma **(H–I)**.

It is well-established that the GINS complex is composed of GINS1/2/3/4 in mammalian cells. First, the Oncomine database was used to assess different mRNA levels of the GINS subunit in SARC patients. We found high GINS1/2/3/4 mRNA expression levels in sarcoma compared to non-tumor tissue (Figure 2). Then, mRNA levels of GINS1/2/3/4 in SARC and non-tumor tissues were detected in TCGA and GTEx databases (Figures 3A–E). The transcriptional levels of GINS1/2/3/4 were significantly higher in SARC than in non-tumor tissues. Meanwhile, the differential expression of GINS genes was verified in the GEO dataset (Figure 3F). From the GSE68591 dataset, SARC samples exhibited significantly high transcriptional levels of GINS1/2/3/4 compared to non-tumor samples. Moreover, the subcellular localization of GINS subunits was explored in the HPA database (Figure 3G). The immunofluorescence imaging results exhibited GINS1/2/3/4 proteins (green) mainly localized to nucleoplasm (blue) in the osteosarcoma cell line (U2OS). Subsequently, the cBioPortal database was used to assess the frequency of gene changes of the GINS subunits in sarcomas. According to these data, GINS1/2/3/4 were altered in 8, 7, 3, and 12 percent of the SARC samples (Figure 3H). Enhanced mRNA transcription was the most frequent GINS subunits alteration in sarcomas (Figure 3I).

### GINS subunits expression in cancer cell lines

**FIGURE 4:**
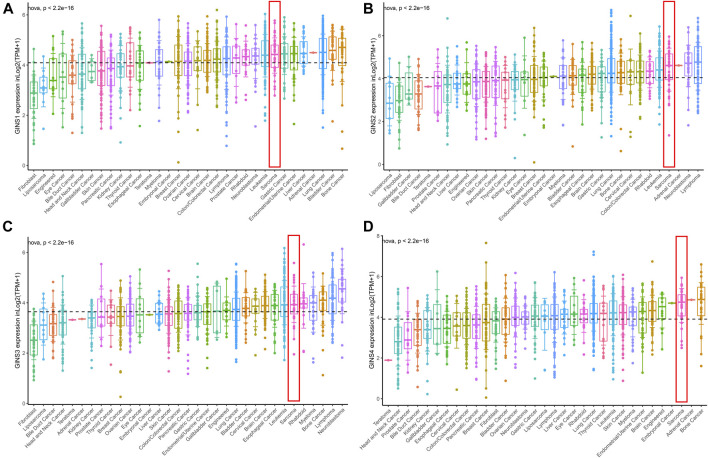
The mRNA expression level of GINS subunits in cancer cell lines from the CCLE database. The expression of GINS1/2/3/4 in SARC ranked eighth, fourth, sixth, and third among various cancer cell lines **(A–D)**.

The CCLE database analysis showed that GINS1/2/3/4 were relatively highly expressed in sarcoma compared to most cancer cell lines. Meanwhile, it was found that at the RNAseq level, GINS1/2/3/4 expression in sarcoma ranked eighth, fourth, sixth and third among various cancer cell lines (Figures 4A–D).

### Prognostic value of GINS subunits in sarcomas patients

**FIGURE 5:**
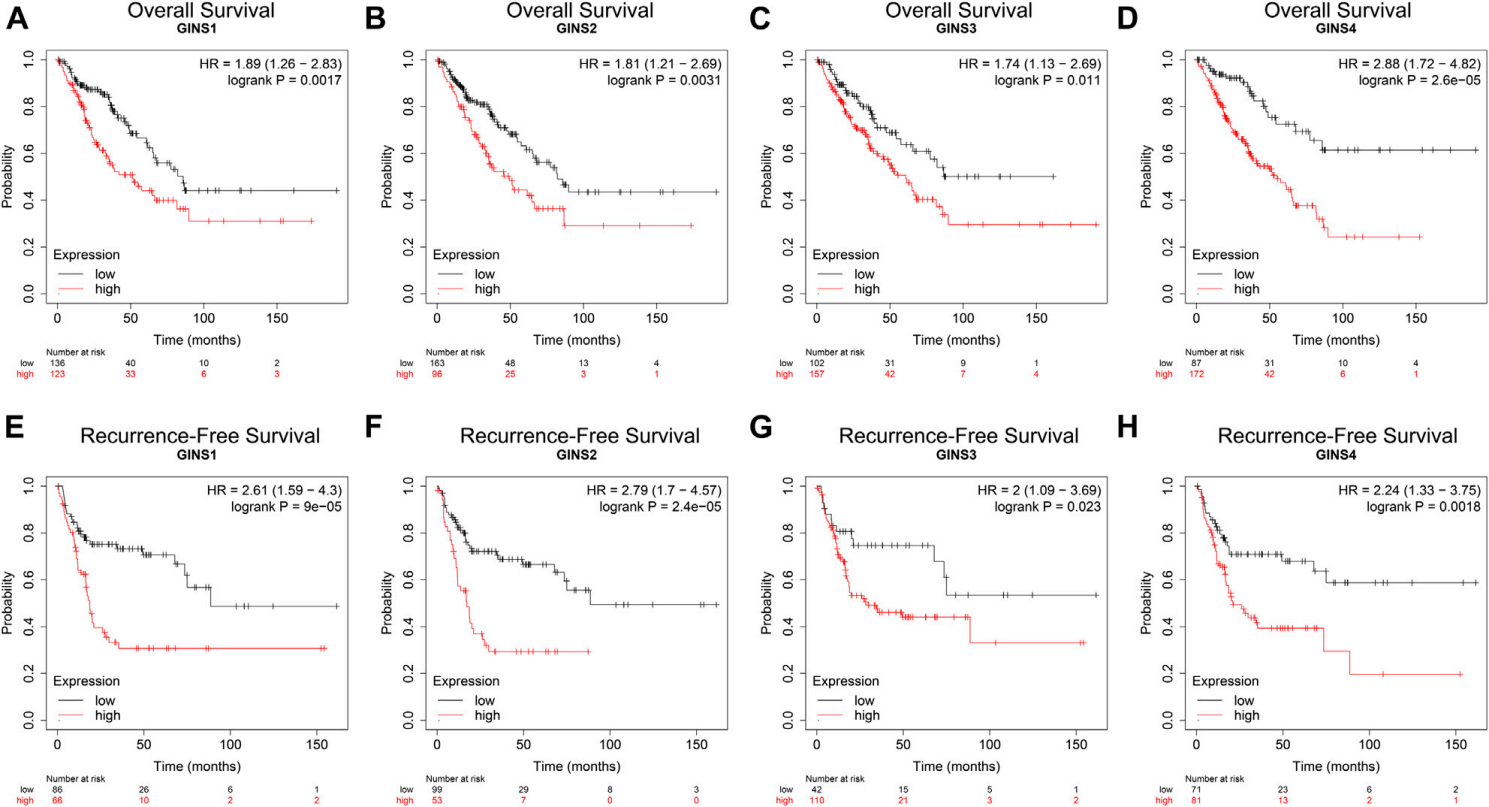
The prognostic value of GINS subunits in sarcoma patients (Kaplan–Meier Plotter). OS curves of GINS1/2/3/4 in SARC **(A–D)**. RFS curves of GINS1/2/3/4 in SARC **(E–H)**.

We used the Kaplan–Meier Plotter Database, GEPIA database, and the R package survival to determine the effect of the GINS gene on the survival of SARC patients. Kaplan–Meier Plotter Database analysis showed that higher expression levels of GINS1/2/3/4 mRNA correlated with lower OS in sarcoma (Figures 5A–D). SARC patients with high GINS1 expression exhibited shorter median OS than those with low GINS1 expression (51.2 vs. 85.83 months; HR = 1.89, *p* = 0.0017). High GINS2 expression in SARC patients was significantly poorer median OS in contrast with low GINS2 expression (49.27 vs. 82.13 months; HR = 1.81, *p* = 0.0031). High GINS3 expression was associated with a shorter upper quartile OS than low GINS3 expression (22.93 vs. 38.07 months; HR = 1.74, *p* = 0.011). Moreover, high GINS4 expression in SARC patients was associated with a poor upper quartile OS in contrast with low GINS4 expressed patients (19.97 vs. 54.23 months; HR = 2.88, *p* = 2.6e-05). In terms of Recurrence-free survival (RFS), high expression of GINS1/2/3/4 predicted a poorer RFS in sarcoma (Figures 5E–H). High GINS1 expression in SARC patients exhibited poorer median RFS compared to low GINS1 expression (18.6 vs. 88.63 months; HR = 2.61, *p* = 9e-05). Moreover, high GINS2 expression in SARC patients correlated with a significantly shorter median RFS in contrast with low GINS2 expression (17.2 vs. 88.63 months; HR = 2.79, *p* = 2.4e-05). High GINS3 expression was associated with a poorer upper quartile RFS compared to low GINS3 expression (11.63 vs. 21.03 months; HR = 2, *p* = 0.023). High GINS4 expression in SARC patients correlated with a shorter upper quartile RFS than low GINS4 expression (10.03 vs. 17.77 months; HR = 2.24, *p* = 0.0018).

**FIGURE 6:**
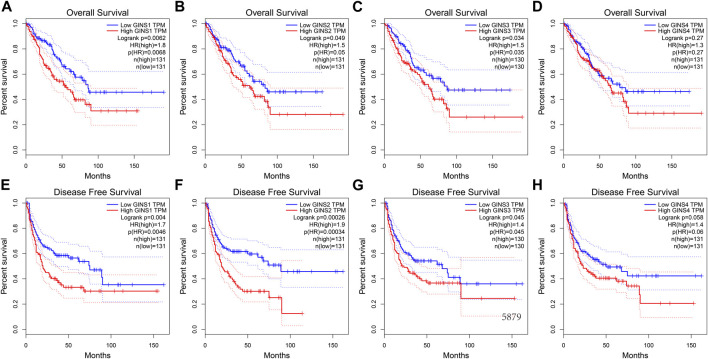
The prognostic value of GINS subunits in sarcoma patients (GEPIA). OS curves of GINS1/2/3/4 in SARC **(A–D)**. DFS curves of GINS1/2/3/4 in SARC **(E–H)**.

Similar results were obtained during the GEPIA database. We found that increased mRNA expression of GINS1/3 in sarcoma was closely related to poorer OS (Figures 6A,C). In this respect, high GINS1 expression in SARC patients correlated with shorter OS than low GINS1 expression (HR = 1.8, *p* = 0.0062). SARC patients with high GINS3 expression had a poorer OS than those with low GINS3 (HR = 1.5, *p* = 0.0035). GINS2 and GINS4 expression in sarcoma also affected the OS, although there was no statistical significance (Figures 6B,D). In terms of Disease-free survival (DFS), high expression of GINS1/2/3 also predicted shorter DFS in sarcoma (Figures 6E–G). We found that high GINS1 expression was associated with poorer DFS than low GINS1 expression (HR = 1.7, *p* = 0.004). The DFS of SARC patients with high GINS2 expression was shorter than those with low GINS2 expression (HR = 1.9, *p* = 0.00026). High GINS3 expression correlated with a poorer DFS compared with low GINS3 expression (HR = 1.4, *p* = 0.045). GINS4 expression was also associated with a relatively poor DFS in sarcoma; however, there was no statistical significance (Figure 6H).

**FIGURE 7:**
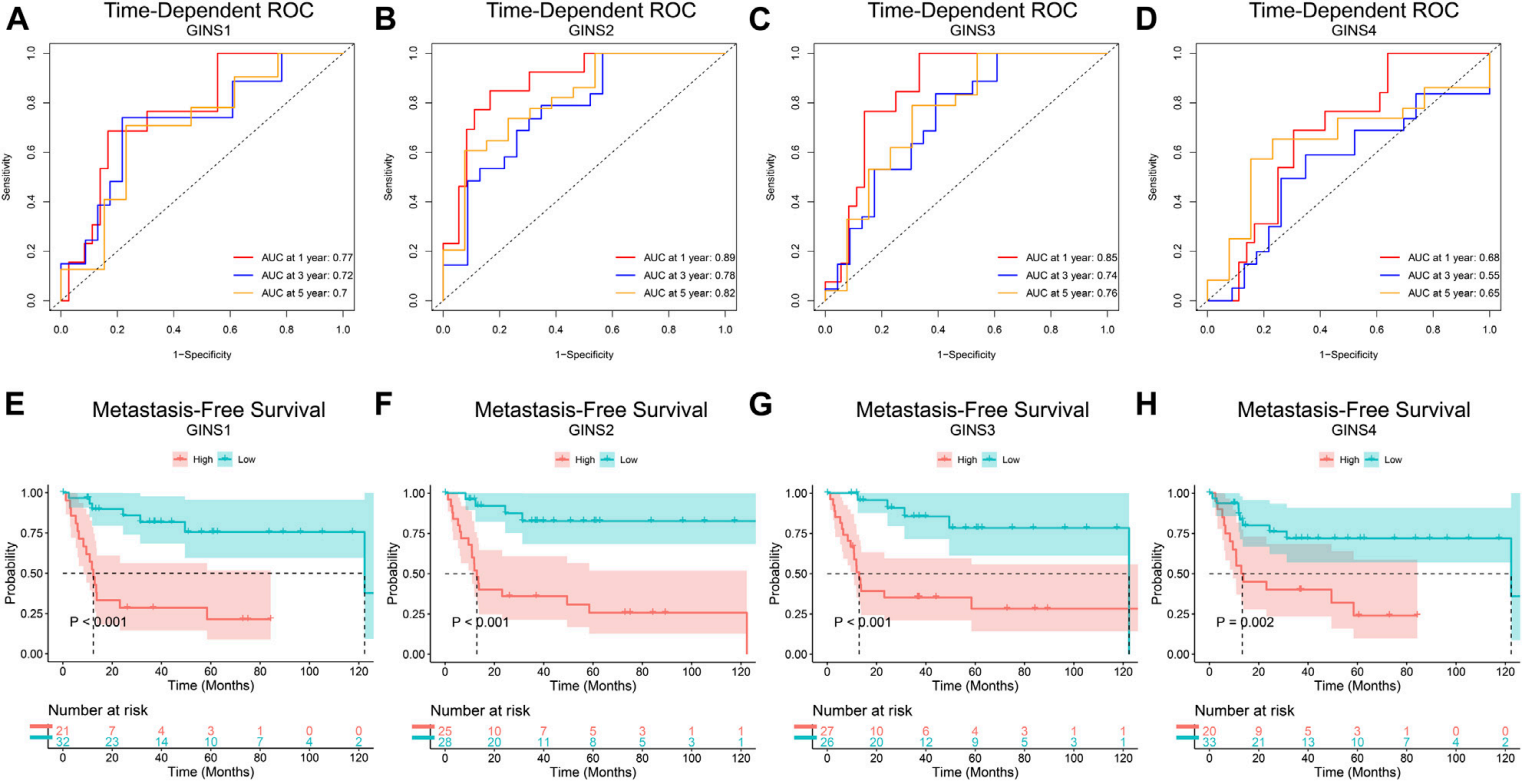
The prognostic value of GINS subunits in sarcoma patients (GSE40021 dataset). Time-dependent ROC curves of GINS1/2/3/4 in SARC **(A–D)**. MFS curves of GINS1/2/3/4 in SARC **(E–H)**.

Finally, the prognostic value of GINS genes was verified in the GEO dataset. ROC analysis of the GSE40021 dataset showed that upregulated GINS1/2/3 expression was closely related to sarcoma metastasis (Figures 7A–C). The AUC values of GINS1 for predicting survival at 1, 3, and 5 years were 0.77, 0.72, and 0.7, while the corresponding AUC values for GINS2 were 0.89, 0.78, and 0.82, respectively. ROC analysis also demonstrated a good predictive value of GINS3 for survival at 1, 3, and 5 years with AUC values of 0.85, 0.74, and 0.76, respectively. The corresponding AUC values for GINS4 were 0.68, 0.55, and 0.65, respectively (Figure 7D). The above results indicate that GINS1/2/3 have prognostic significance in predicting sarcoma metastasis. Furthermore, Kaplan-Meier analysis of metastasis-free survival (MFS) of GINS subunits was performed in the GSE40021 dataset (Figures 7E–H). SARC patients with high GINS1 expression exhibited shorter mean MFS than patients with low GINS1 expression (28.95 vs. 104.17 months; *p* = 2.00e-05). The mean MFS of SARC patients with high GINS2 expression was poorer than those with low GINS2 expression (42.85 vs. 116.59 months; *p* = 3.03e-05). Moreover, patients with high GINS3 expression had a shorter mean MFS than those with low GINS3 expression (48.71 vs. 102.85 months; *p* = 0.00025). Finally, SARC patients with high GINS4 expression had a poor mean MFS compared with patients with low GINS4 expression (34.49 vs. 97.30 months; *p* = 0.0018).

### The co-expression and interaction analyses of GINS subunits in sarcomas patients

**FIGURE 8:**
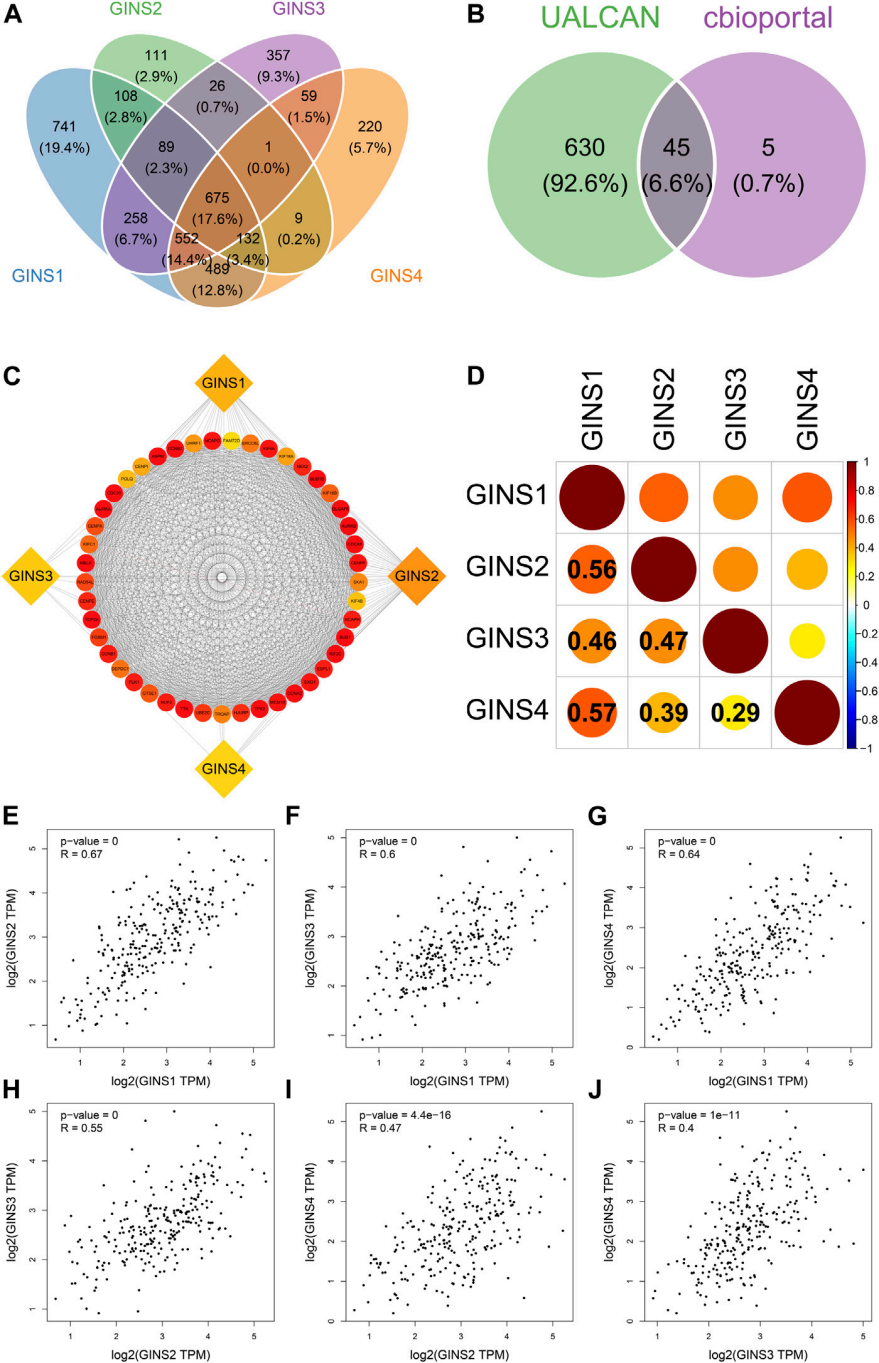
Co-expressed genes and co-expression analysis of GINS subunits in SARC. Common 675 genes among co-expressed genes of GINS1/2/3/4 analyzed by UALCAN **(A)**. 45 co-expressed genes simultaneously predicted by UALCAN and cBioPortal database **(B)**. The protein-protein interaction analysis of GINS1/2/3/4 and 45 co-expressed genes **(C)**. The co-expression analysis of GINS1/2/3/4 in SARC from the cBioPortal database **(D)**. The co-expression analysis of GINS1/2/3/4 in SARC from the GEPIA database **(E–J)**.

Our comprehensive analysis identifed 675 genes and the top 50 most frequently genes co-expressed with GINS1/2/3/4 in sarcoma by UALCAN and cBioPortal database, respectively (Figure 8A). Finally, 45 co-expressed genes were simultaneously predicted by these two databases (Figure 8B). These data suggest that EXO1, UHRF1, DLGAP5, MCM10, ESPL1, TPX2, NCAPH, CENPI, ERCC6L, CCNA2, POLQ, TTK, AURKA, BUB1, KIF18B, CDCA8, KIF4A, KIF4B, NCAPG, CENPA, CENPE, CCNB1, KIF2C, FOXM1, CCNB2, HJURP, KIF18A, BUB1B, NEK2, DEPDC1, CENPF, PLK1, TROAP, KIFC1, ASPM, CDC20, TOP2A, NUF2, SKA1, FAM72D, MELK, UBE2C, AURKB, RAD54L, and GTSE1 were closely related to the biological functions and signaling pathways of differentially expressed GINS subunits in SARC. The protein-protein interaction results showed that GINS1/2/3/4 subunits and 45 co-expressed genes were closely related (Figure 8C). In addition, the cBioPortal and GEPIA databases were used to analyze the co-expression relationships between different GINS genes. Analysis in the cBioPortal database showed GINS1 was positively correlated with GINS2/3/4, with Pearson correlation coefficient (r) values of 0.56, 0.46, and 0.57, respectively (P_s_ < 0.05). GINS2 was significantly correlated with GINS3 and GINS4, with r values of 0.47 and 0.39, respectively (P_s_ < 0.05). GINS3 was significantly correlated with GINS4 with a correlation coefficient value of 0.29 (*p* < 0.05) (Figure 8D). Based on GEPIA database analysis, similar results were found. GINS1 was positively associated with GINS2/3/4, with r values of 0.67, 0.6, and 0.64, respectively (P_s_ < 0.05) (Figures 8E–G). GINS2 was significantly associated with GINS3 and GINS4, with r values of 0.55 and 0.47, respectively (P_s_ < 0.05) (Figures 8H,I). GINS3 was correlated with GINS4 with a correlation coefficient value of 0.4 (*p* < 0.05) (Figure 8J).

### Functional enrichment analysis of GINS subunits in sarcomas

**FIGURE 9:**
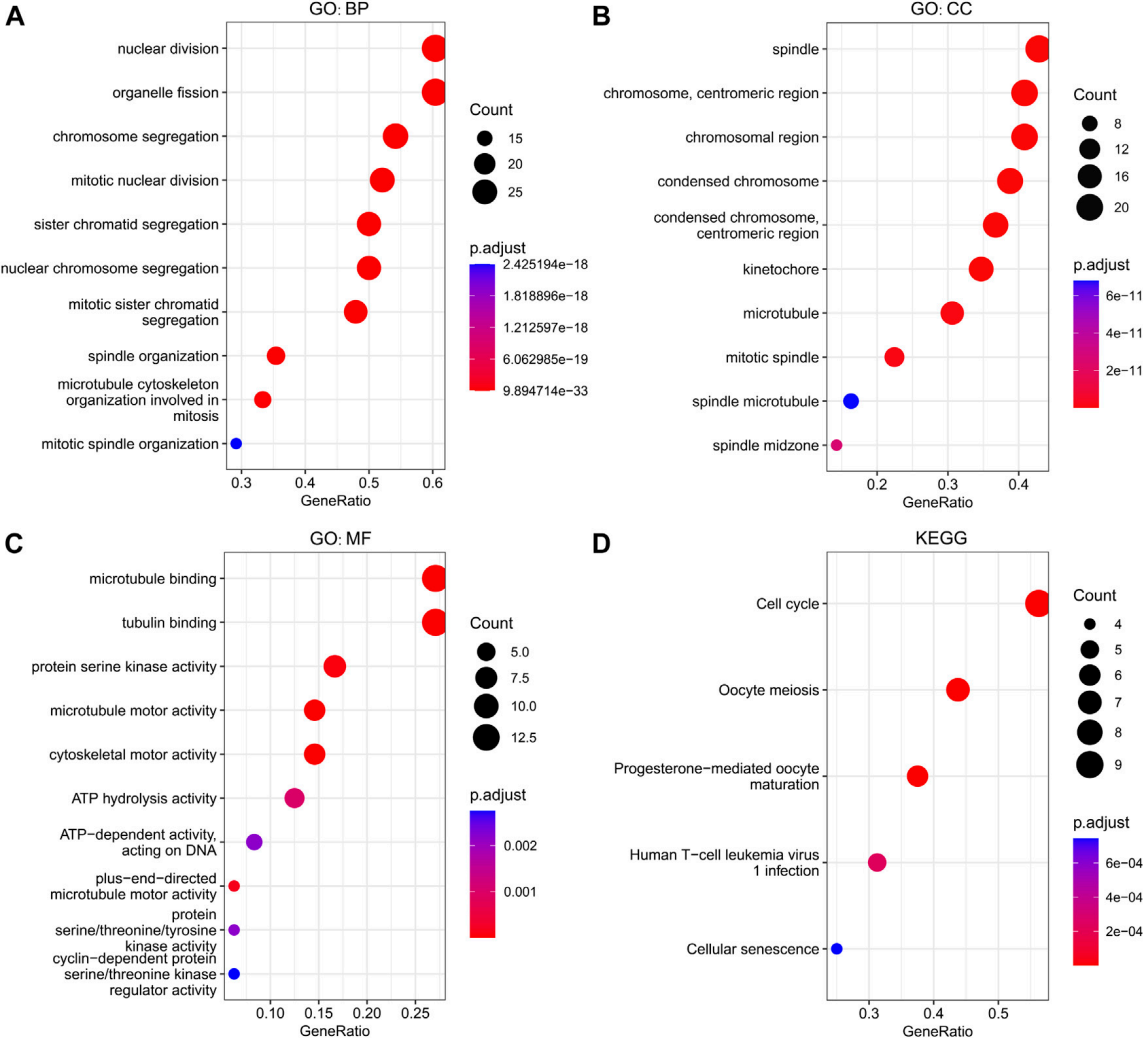
Functional enrichment analysis of GINS genes and their neighboring genes in SARC. Biological process **(A)**, Cellular component **(B)**, Molecular function **(C)**, and KEGG pathway **(D)** related to GINS1/2/3/4 and their neighboring genes in SARC.

The GINS genes and their neighboring genes in SARC were analyzed by R package clusterProfiler for GO and KEGG enrichment analysis. These genes were found primarily involved in chromosome segregation and cell division, associated with the genesis and progression of malignant tumors. Consistent with the literature, in the BP category, these genes were significantly enriched in the mitosis, spindle, and microtubule cytoskeleton (Figure 9A). The spindle, chromosome centromeric region, chromosomal region, condensed chromosome, condensed chromosome centromeric region, kinetochore, microtubule, mitotic spindle, spindle microtubule, and spindle midzone were the top ten most significantly enriched items in the CC category (Figure 9B). In terms of MF, the GO terms were significantly enriched in processes, including microtubule binding, protein serine/threonine/tyrosine kinase activity, microtubule motor activity, cytoskeletal motor activity, ATP hydrolysis activity, and cyclin−dependent protein serine/threonine kinase regulator activity (Figure 9C). The KEGG pathway analysis showed that GINS genes and their neighboring genes were remarkably enriched in cell cycle, oocyte meiosis, progesterone-mediated oocyte maturation, human T-cell leukemia virus one infection, and cellular senescence (Figure 9D). More detailed information is shown in Table 1.

**TABLE 1:** GO and KEGG enrichment analysis of GINS genes and their neighboring genes.

Category	ID	Description	Count	*p*-value
BP	GO:0000280	Nuclear division	29	1.65E-35
BP	GO:0048285	Organelle fission	29	3.72E-34
BP	GO:0007059	Chromosome segregation	26	6.34E-33
BP	GO:0140014	Mitotic nuclear division	25	3.41E-33
BP	GO:0000819	Sister chromatid segregation	24	3.92E-35
BP	GO:0098813	Nuclear chromosome segregation	24	1.48E-31
BP	GO:0000070	Mitotic sister chromatid segregation	23	4.44E-35
BP	GO:0007051	Spindle organization	17	1.14E-22
BP	GO:1902850	Microtubule cytoskeleton organization involved in mitosis	16	1.64E-22
BP	GO:0007052	Mitotic spindle organization	14	3.63E-20
CC	GO:0005819	Spindle	21	2.87E-23
CC	GO:0000775	Chromosome, centromeric region	20	1.06E-27
CC	GO:0098687	Chromosomal region	20	1.05E-22
CC	GO:0000779	Condensed chromosome, centromeric region	18	1.29E-26
CC	GO:0000779	Condensed chromosome, centromeric region	18	1.29E-26
CC	GO:0000776	Kinetochore	17	3.08E-25
CC	GO:0005874	Microtubule	15	8.24E-14
CC	GO:0072686	Mitotic spindle	11	1.86E-13
CC	GO:0005876	Spindle microtubule	8	9.06E-12
CC	GO:0051233	Spindle midzone	7	3.14E-12
MF	GO:0008017	Microtubule binding	13	1.43E-13
MF	GO:0015631	Tubulin binding	13	8.81E-12
MF	GO:0106310	Protein serine kinase activity	8	3.83E-06
MF	GO:0003777	Microtubule motor activity	7	5.03E-10
MF	GO:0003774	Cytoskeletal motor activity	7	1.76E-08
MF	GO:0016887	ATP hydrolysis activity	6	7.27E-05
MF	GO:0008094	ATP-dependent activity, acting on DNA	4	2.00E-04
MF	GO:0008574	Plus-end-directed microtubule motor activity	3	1.11E-05
MF	GO:0004712	Protein serine/threonine/tyrosine kinase activity	3	2.06E-04
MF	GO:0016538	Cyclin-dependent protein serine/threonine kinase regulator activity	3	3.01E-04
KEGG	hsa04110	Cell cycle	9	3.91E-13
KEGG	hsa04114	Oocyte meiosis	7	2.37E-09
KEGG	hsa04914	Progesterone-mediated oocyte maturation	6	2.38E-08
KEGG	hsa05166	Human T-cell leukemia virus 1 infection	5	4.87E-05
KEGG	hsa04218	Cellular senescence	4	1.96E-04

### The ceRNA network of GINS subunits in sarcomas

**FIGURE 10:**
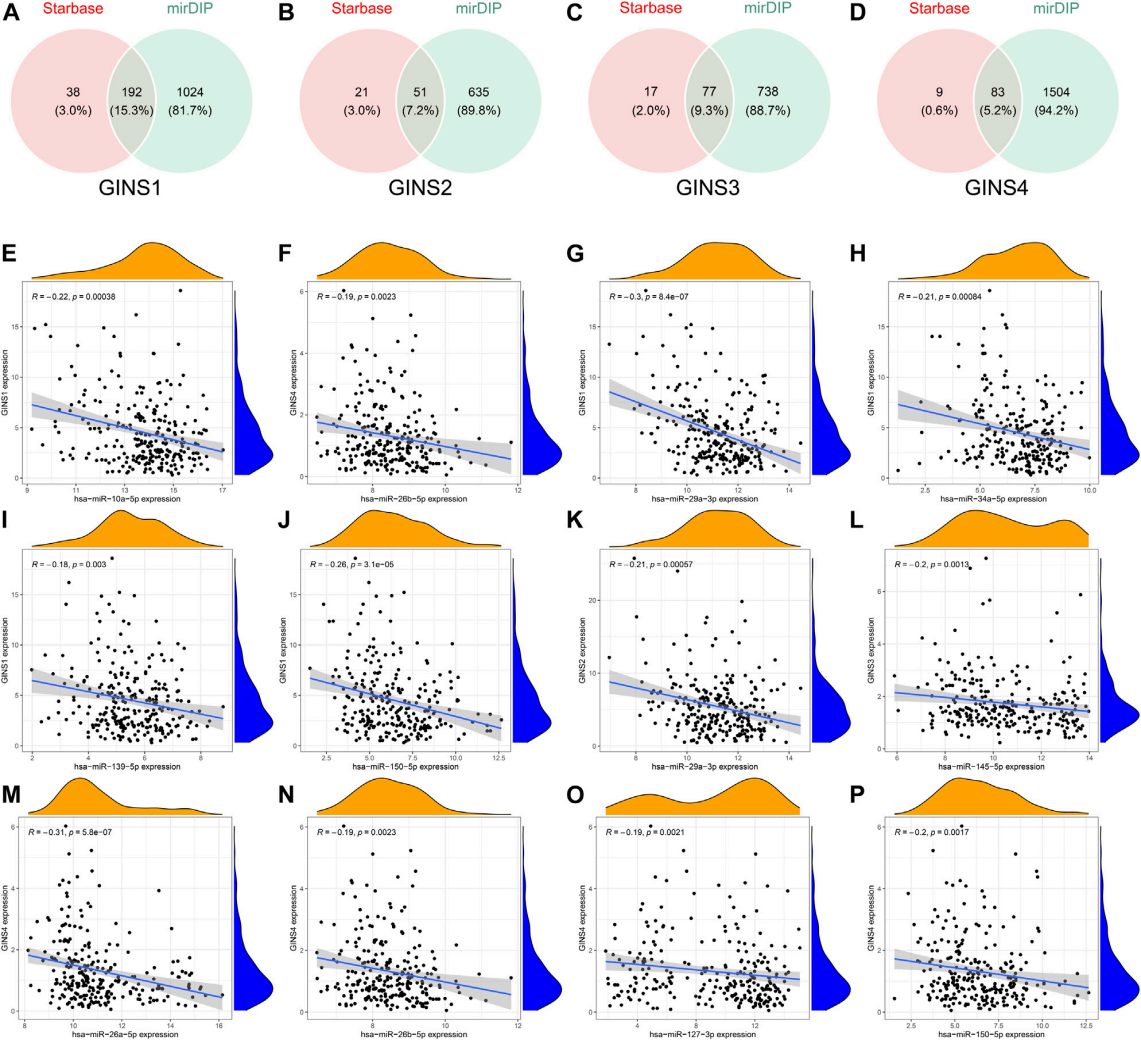
Comprehensive analysis of miRNAs targeting GINS1/2/3/4 in SARC. Venn graphs illustrating common miRNAs predicted to target GINS1/2/3/4 by Starbase and mirDIP **(A–D)**. The miRNAs negatively co-expressed with GINS1/2/3/4 **(E–P)**.

**FIGURE 11:**
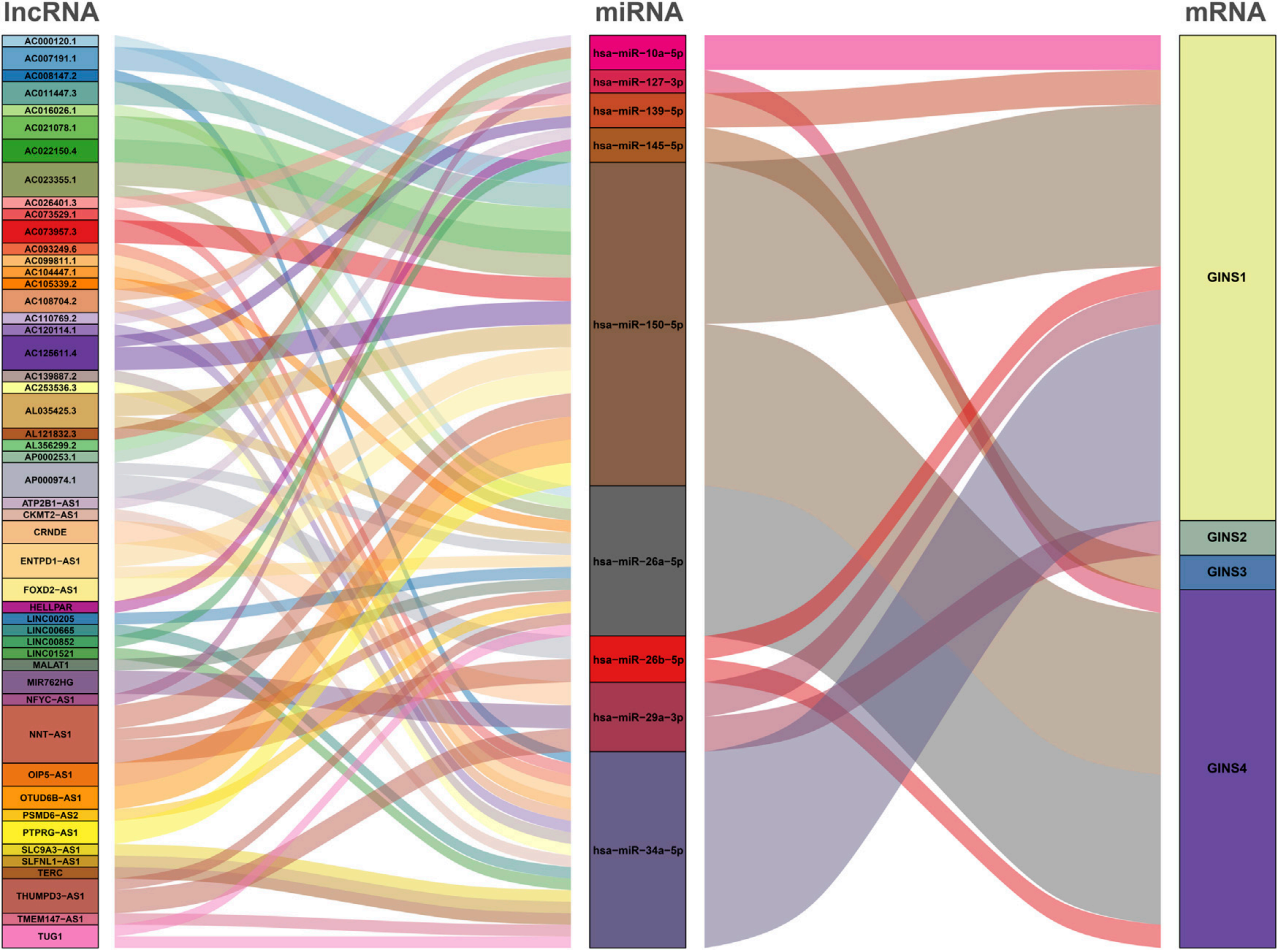
Sankey diagram of the ceRNA network targeting GINS1/2/3/4 in SARC.

**FIGURE 12:**
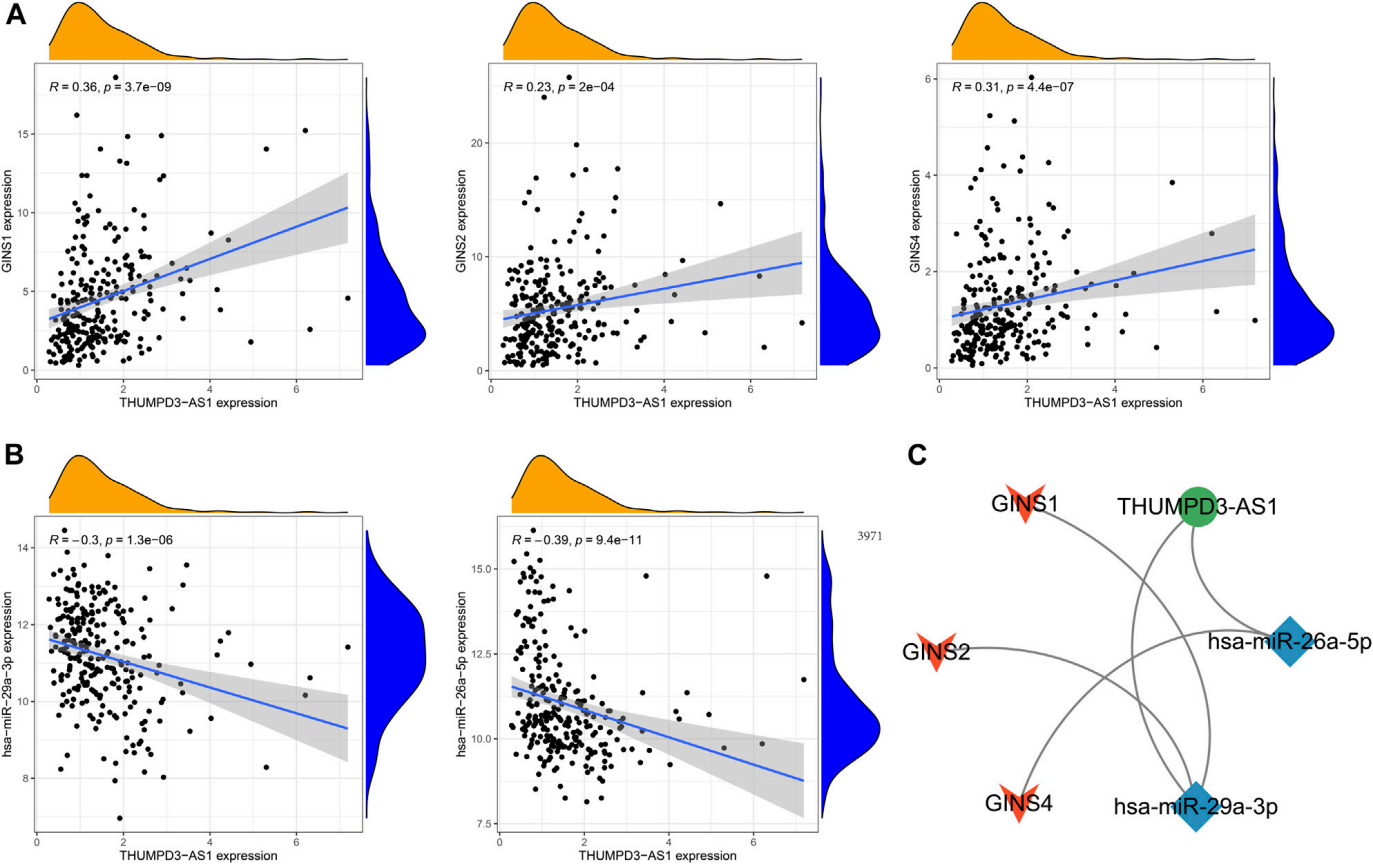
The sub-network of lncRNA THUMPD3-AS1, miRNA and GINS subunits in SARC. The GINS subunits positively coexpressed with THUMPD3-AS1 **(A)**. The miRNAs negatively co-expressed with THUMPD3-AS1 **(B)**. The network diagram demonstrated a sub-network of THUMPD3-AS1-miRNA-GINS **(C)**.

The GINS gene-related ceRNA networks were predicted and constructed using online tools and Rstudio based on TCGA-SARC dataset. Through the starBase and mirDIP websites, 230 and 1216 miRNAs were predicted to target GINS1, respectively, of which 192 miRNAs were simultaneously predicted by both websites (Figure 10A). Meanwhile, the co-expression analysis of these 192 miRNAs in TCGA-SARC dataset illustrate that hsa-miR-10a-5p, hsa-miR-26b-5p, hsa-miR-29a-3p, hsa-miR-34a-5p, hsa-miR-139-5p and hsa-miR-150-5p were negatively co-expressed with GINS1, with R values of −0.22, −0.18, −0.3, −0.21, −0.18 and −0.26, respectively (P_s_ < 0.005) (Figures 10E–J). Moreover, the starBase and mirDIP websites predicted the binding of 72 and 686 miRNAs to GINS2, respectively, of which 51 were predicted by both websites (Figure 10B). The co-expression analysis of these 51 miRNAs in TCGA-SARC dataset showed that only hsa-miR-29a-3p was negatively correlated to the transcriptional levels of GINS2, with the r values of −0.21 (*p* = 0.00057) (Figure 10K). In addition, we predicted 94 and 815 miRNAs targeting GINS3 using starBase and mirDIP websites, respectively, of which 77 miRNAs were predicted by both websites (Figure 10C). Then, a correlation analysis of these 77 miRNAs expression in TCGA-SARC dataset showed that only hsa-miR-145-5p was negatively co-expressed with GINS3, with the r values of −0.2 (*p* = 0.0013) (Figure 10L). Furthermore, starBase and mirDIP websites predicted 92 and 1587 miRNAs were bound to GINS4, respectively, of which 83 miRNAs were predicted by both websites (Figure 10D). The co-expression analysis of these 83 miRNAs in TCGA-SARC dataset showed that has-miR-26a-5p, has-miR-26b-5p, has-miR-127-3p, and has-miR-150-5p were negatively correlated with the transcriptional levels of GINS4, with the r values −0.31, −0.19, −0.19 and −0.2, respectively (P_s_ < 0.005) (Figures 10M–P). Subsequently, starBase predicted 77, 92, 92, 114, 125, 28, 75, 113 and 187 lncRNAs were bound to has-miR-10a-5p, has-miR-26a-5p, has-miR-26b-5p, has-miR-29a-3p, has-miR-34a-5p, has-miR-127-3p, has-miR-139-5p, has-miR-145-5p, and has-miR-150-5p, respectively. Co-expression analysis in TCGA-SARC dataset found that AC110769.2, AL356299.2, and AL121832.3 were found to be negatively co-expressed with has-miR-10a-5p (r = −0.22, −0.22 and −0.32) and positively correlated with the transcriptional levels of GINS1 (r = 0.3, 0.36 and 0.48). Moreover, AC016026.1, THUMPD3-AS1, LINC00205, ENTPD1-AS1, PSMD6-AS2, AC000120.1, NNT-AS1, MALAT1, AC105339.2, TUG1, AC023355.1, AL035425.3 and AP000974.1, were found to be negatively co-expressed with hsa-miR-26a-5p (r = −0.3, −0.39, −0.23, −0.21, −0.21, −0.22, −0.37, −0.32, −0.25, −0.34, −0.26, −0.22, and −0.33) and positively correlated with the transcriptional levels of GINS4 (r = 0.24, 0.31, 0.35, 0.26, 0.28, 0.26, 0.29, 0.23, 0.39, 0.39, 0.25, 0.21, and 0.3). Furthermore, NNT-AS1 and AP000974.1 were found to be negatively co-expressed with hsa-miR-26b-5p (r = −0.31 and −0.25) and positively correlated with the transcriptional levels of GINS1 (r = 0.3 and 0.28) and GINS4 (r = 0.29 and 0.3). In addition, THUMPD3-AS1, CRNDE, and MIR762HG were found to be negatively co-expressed with hsa-miR-29a-3p (r = −0.3, −0.28 and −0.26) and positively correlated with the transcriptional levels of GINS1 (r = 0.36, 0.28 and 0.3) and GINS2 (r = 0.23, 0.31 and 0.31). Subsequently, we found that LINC01521, SLC9A3-AS1, AC253536.3, LINC00665, AC073529.1, TMEM147-AS1, AC099811.1, CKMT2-AS1, AC120114.1, AC139887.2, TUG1, AC008147.2, AC093249.6, TERC, AC108704.2, AC104447.1 and SLFNL1-AS1 were negatively co-expressed with hsa-miR-34a-5p (r = −0.32, −0.27, −0.37, −0.25, −0.31, −0.24, −0.41, −0.41, −0.45, −0.27, −0.38, −0.23, −0.27, −0.21, −0.34, −0.26, and −0.45) and positively correlated with the transcriptional levels of GINS1 (r = 0.36, 0.22, 0.22, 0.34, 0.56, 0.26, 0.21, 0.22, 0.48, 0.22, 0.33, 0.23, 0.25, 0.21, 0.21, 0.36, and 0.32). Additionally, AP000253.1 and NFYC-AS1 were negatively co-expressed with hsa-miR-127-3p (r = −0.28 and −0.46) and positively correlated with the transcriptional levels of GINS4 (r = 0.23 and 0.3); AC125611.4, AC108704.2 and AC026401.3 were found to be negatively co-expressed with hsa-miR-139-5p (r = −0.29, −0.21 and −0.26) and positively correlated with the transcriptional levels of GINS1 (r = 0.4, 0.21 and 0.39). LINC00852, ATP2B1-AS1 and HELLPAR were found to be negatively co-expressed with hsa-miR-145-5p (r = −0.28, −0.39 and −0.24) and positively correlated with the transcriptional levels of GINS3 (r = 0.29, 0.24 and 0.21). Finally, ENTPD1-AS1, AC021078.1, FOXD2-AS1, PTPRG-AS1, OIP5-AS1, NNT-AS1, OTUD6B-AS1, AC125611.4, AC023355.1, AL035425.3, AC011447.3, AC022150.4, AC073957.3, and AC007191.1 were found to be negatively co-expressed with hsa-miR-150-5p (R = −0.24, −0.21, −0.23, −0.40, −0.32, −0.28, −0.24, −0.39, −0.22, −0.41, −0.31, −0.26, −0.25, and −0.37) and positively correlated with the transcriptional levels of GINS1 (r = 0.27, 0.21, 0.37, 0.35, 0.24, 0.30, 0.29, 0.40, 0.30, 0.27, 0.41, 0.42, 0.24, and 0.31) and GINS4 (r = 0.26, 0.27, 0.25, 0.25, 0.31, 0.20, 0.29, 0.25, 0.31, 0.25, 0.21, 0.36, 0.52, 0.35, and 0.38) (P_s_ < 0.005). The interactive relationship of the ceRNA network is shown in a Sankey diagram (Figure 11). Finally, THUMPD3-AS1 was found to be a lncRNA that regulates most mRNAs of GINS genes (GINS1/2/4) in sarcoma by binding to hsa-miR-26a-5p and hsa-miR-29a-3p simultaneously (Figures 12A,B). A sub-network of lncRNA THUMPD3-AS1, miRNA and GINS subunits in SARC was constucted by Cytoscape software (Figure 12C).

### Immune cell infiltration of GINS subunits in patients with sarcomas

**FIGURE 13:**
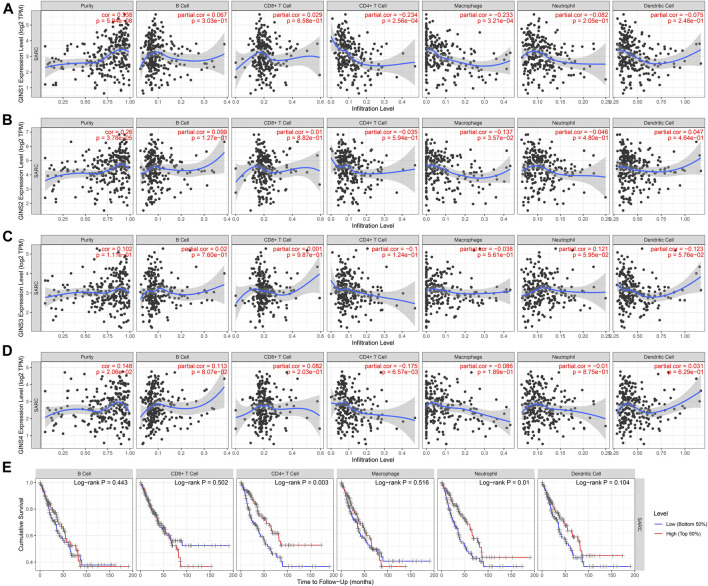
Correlation of immune infiltration level with GINS1/2/3/4 expression in SARC **(A–D)**. Meanwhile, high CD4+ T cell and neutrophil infiltration levels were associated with better survival outcomes in SARC **(E)**.

The TIMER database was utilized to detect the expression of GINS genes with tumor purity and immune cell infiltration and the effect of immune cell infiltration on survival in SARC. We found that GINS1 expression was positive correlated with tumor purity (r = 0.338, *p* = 5.94e-08), and negative correlated with the levels of tumor-infiltrating CD4+ T cell (r = −0.234, *p* = 2.56e-04) and macrophages (r = −0.233, *p* = 3.21e-04) (Figure 13A). GINS2 was positive correlated with tumor purity (r = 0.26, *p* = 3.78e-05), and negative correlated with the levels of tumor-infiltrating macrophages (r = −0.137, *p* = 3.57e-02) (Figure 13B). GINS3 was positive correlated with tumor purity (r = 0.102, *p* = 1.11e-01) (Figure 13C). Finally, GINS4 was positive correlated with tumor purity (r = 0.148, *p* = 2.06e-02), and negative correlated with the levels of tumor-infiltrating CD4+ T cell (r = −0.175, *p* = 6.57e-03) (Figure 13D). Meanwhile, high CD4+ T cell and neutrophil infiltration levels were associated with better survival outcomes in SARC (*p* < 0.05) (Figure 13E). These findings suggest that GINS genes may influence patient survival by interacting with the infiltrated immune cells in sarcomas.

## Discussion

The present study explored the role of GINS subunits in SARC from several perspectives, including mRNA transcription levels, disease survival, tumor immunization, biological functions and signaling pathways analysis.

The GINS complex, which consists of four subunits of GINS1/2/3/4, is well-established as the core structure of CMG helicase and regulates the formation of DNA replication forks and chromosome replication (Li and O’Donnell, 2018). Abnormal replication of DNA can affect the cell cycle and proliferation, leading to cancer or other diseases that promote cancer. Therefore, as an essential regulator of DNA replication, GINS complex subunits can be considered a potential biological target for diagnosing and treating malignant tumors (Seo and Kang, 2018). Current evidence suggests that GINS subunit expression is upregulated in various malignant tumors and closely related to prognosis. Nonetheless, the prognostic value, molecular mechanisms and ceRNA network of GINS subunits in SARC remain largely understudied. To the best of our knowledge, this is the first in silico analysis to investigate the specific effects of all GINS subunits as a whole in SARC.

It has been established that GINS1 plays a crucial role in forming the CMG complex by close contact with GINS tetramer through its C-terminal B domain. The GINS1 subunit, known as Psf1, is a 22988 Da protein composed of 196 amino acids, and its encoding gene is located on chromosome 20P11.21. Overwhelming evidence substantiates that up-regulation of GINS1 expression in HCC tumors is correlated with tumor grade. In this regard, the knockout of GINS1 reportedly leads to cell cycle arrest in the G1/S phase, thereby reducing the proliferation of tumor cells (Li et al., 2021a). Tang et al. (2019) found that the upregulated expression of GINS1 in synovial sarcoma was associated with shorter survival, and inhibition of GINS1 expression can result in restricted proliferation and even apoptosis of synovial sarcoma cells. Our results showed that GINS1 transcription was significantly elevated in sarcoma tissue, and GINS1 was overexpressed in sarcoma cell lines. Moreover, increased GINS1 expression in SARC correlated with the poor OS and DFS.

Moreover, GINS2 regulates the cell cycle and proliferation through a series of receptor and growth factor interactions. The GINS2 subunit, known as Psf2, is a 21428 Da protein composed of 185 amino acids, and its encoding gene is located on chromosome 16q24.1. The GINS complex is in extensive contact with Cdc45 through the A domain of the GINS2 subunit. For example, a previous study found that inhibiting GINS2 expression promotes apoptosis of thyroid cancer cells by mediating the down-regulation of downstream proteins CITED2 and LOXL2 (Ye et al., 2019). Yan et al. (2018) identified that GINS2 mRNA transcription was upregulated in ovarian cancer cells while inhibiting GINS2 expression reduced the proliferation and viability of ovarian cancer cells by interfering with the cell cycle. Meanwhile, Huang L et al. found that GINS2 could activate the ERK/MAPK signaling to promote epithelial-mesenchymal transformation (EMT) in pancreatic cancer (Zhang et al., 2020b). Consistent with the literature, our study found that GINS2 was upregulated in sarcomas and overexpressed in sarcoma cell lines. Similarly, the increased GINS2 expression in sarcoma patients was associated with poor OS and DFS.

Furthermore, GINS3 binds the GINS complex to the N-terminal domain of MCM3 by contact below the A-domain. The GINS3 subunit, known as Psf3, is a 24535 Da protein composed of 216 amino acids, and its encoding gene is located on chromosome 16q21. Tane S et al. found that in GINS3 silenced NSCLC cell lines, the proportion of the S stage was significantly reduced, resulting in inhibition of proliferative activity (Tane et al., 2015). Hokka et al. (2013) found that increased GINS3 expression played a vital role in lung adenocarcinoma progression and predicted a shorter survival time for primary lung adenocarcinoma patients. Herein, we revealed that GINS3 mRNA transcription was significantly elevated in sarcoma tissues and cell lines. Furthermore, increased GINS3 expression correlated with a shorter OS and DFS in sarcomas.

Finally, GINS4 is the first subunit of the GINS tetramer isolated from eukaryotes. GINS4 stabilizes GINS tetramer by inserting its C-terminal B domain between the two A domains of GINS4 and GINS2. The GINS4 subunit, known as Sld5, is a 26047 Da protein composed of 223 amino acids, and its encoding gene is located on chromosome 8p11.21. This GINS complex subunit is closely related to the normal cell cycle and replication. Rong et al. (2020) identified GINS4 as an important prognostic biomarker that promotes colorectal cancer growth by inhibiting apoptosis and accelerating cell cycle and colony formation processes. Liu et al. (2021) found that GINS4 was upregulated in gliomas and associated with poorer survival, and may regulate the immune microenvironment and promote the malignant progression of gliomas by participating in the JAK-STAT pathway. In the present study, we also found that GINS4 was upregulated in sarcoma tissues and cell lines and was associated with shorter OS and DFS.

Given that multiple GINS subunits are upregulated in sarcomas, their genetic changes were further explored in our research. GINS subunits that were differentially expressed in sarcomas exhibited many genetic alterations. Mutation analysis revealed various genetic changes involving GINS subunits in SARC. These results indicated that upregulated mRNA expression is the primary genetic alteration of all GINS subunits in SARC. The pathogenesis of sarcomas is widely thought to be multifactorial, and genetic changes play an essential role in this process.

In our research, a weak to strong positive correlation was found between the differentially expressed GINS subunits, revealing that these genes promote each other in mediating the occurrence and development of sarcoma. Afterward, the effects of mutations in the target gene and the related 50 neighboring genes on the potential biological functions and signaling pathways of sarcoma patients were analyzed by KEGG pathway and GO enrichment analysis. As expected, the function of these genes was found to be primarily related to the cell cycle, human T-cell leukemia virus one infection (HTLV-1), and cellular senescence. It is widely acknowledged that the cell cycle is a conserved evolutionary process critical to cell growth (Huber et al., 2021). Despite multiple mechanisms controlling the cell cycle, the formation of the CMG complex is one of the most crucial regulatory processes in the eukaryotic cell cycle (Parker et al., 2017). It has been shown that dysregulation of the cell cycle can lead to abnormal cell proliferation and tumorigenesis (Stewart et al., 2003). HTLV-1 signaling promotes tumor formation by encoding regulatory proteins such as Tax and HBZ that activate the cyclin-dependent kinases, nuclear factor -κB, and Akt signaling pathways, and silence p53 function (Mesri et al., 2014; Pierangeli et al., 2015). Cell senescence is initiated by the up-regulation of proto-oncogene expression or inhibition of tumor suppressor genes. (Wei and Ji, 2018). Senescent cells promote malignant tumor progression and invasion in the tumor microenvironment by releasing senescence-related secretory phenotype (SASP) (Prieto and Baker, 2019; Birch and Gil, 2020). Taken together these findings suggest that GINS subunits are promising molecular therapeutic targets in sarcomas.

In recent years, the ceRNA network has been documented as a novel RNA interaction mechanism and is considered one of the critical components of post-transcriptional regulation (Su et al., 2021). This hypothesis is based on the theory that miRNAs can bind corresponding target mRNAs to prevent translation or their degradation, whereas the ceRNA can competitively bind specific miRNA through miRNA reaction elements (MRE) to regulate miRNA-targeted mRNA expression (Qi et al., 2020). Our research comprehensively analyzed and constructed the lncRNA-miRNA-mRNA interacted ceRNA network involving GINS genes in sarcoma. In this ceRNA network analysis, three miRNAs, hsa-miR-26b-5p, hsa-miR-29a-3p, and hsa-miR-150-5p, were observed to target multiple GINS mRNAs simultaneously, while the THUMPD3-AS1 was found to be a lncRNA that regulates most mRNAs of GINS genes in sarcoma by binding simultaneously to hsa-miR-26a-5p and hsa-miR-29a-3p. An increasing body of evidence from recently published studies suggests that hsa-miR-26a-5p (Fan et al., 2022), hsa-miR-26b-5p (Tang et al., 2022), hsa-miR-29a-3p (Pan et al., 2021), and hsa-miR-150-5p (Li et al., 2021b) can influence the oncogenesis and progression of various tumors by participating in lncRNA-miRNA-mRNA interactions as protective factors. THUMPD3-AS1 was found to act as a ceRNA to block the effects of miRNAs such as miR-543 and miR-218 to promote tumor cell proliferation and self-renewal (Hu et al., 2019; Pu et al., 2022). However, the role of these non-coding RNAs in sarcomas remains largely understudied. Importantly, the present study provides potential directions for future research.

With the development of medical technology, the principles of effective treatment for sarcomas have become apparent, but survival outcomes for patients with advanced sarcomas have not improved significantly (HaDuong et al., 2015). Tumor immunotherapy has become a new therapeutic method in addition to traditional surgery, chemotherapy and radiotherapy by activating the host’s natural defense system to recognize and destroy tumor cells (Li et al., 2020a). It is a compelling emerging treatment that has shown survival benefits in many types of cancer (Chiang and Kandalaft, 2018). The tumor microenvironment (TME) is composed of cancer cells and adjacent normal cells and has a crucial impact on the proliferation and invasion of malignant tumors (Quail and Joyce, 2013). Tumor immune cell infiltration is closely related to malignant behaviors such as angiogenesis, tumorigenesis, and metastasis and thus regulates the number and differentiation of immune cells in TME (Scott et al., 2021). Current evidence suggests that the imbalance between tumor and host immune response may cause tumor progression (Lorusso and Ruegg, 2008). Therefore, it is crucial to clarify the immune status of TME, including the number and phenotype of infiltrating immune cells, for improving immunotherapy response and clinical outcomes (Huff et al., 2019). This study explored six types of infiltrated immune cells in SARC tissues and surrounding non-tumor tissues and analyzed the effect of GINS subunits on immune cell infiltration. The results substantiated that sarcoma patients with elevated CD4+ T cell and neutrophil infiltration levels have better survival outcomes. Interestingly, it has been confirmed that CD4+ T cells can cause cancer cell death through ferroptosis and the contact killing mechanism of anti-MHC CLASS II antibodies *in vitro* (Oh et al., 2020). Few studies have hitherto documented the immunosuppressive antitumor behavior of neutrophils, which may be due to the current lack of research in this area, rather than neutrophils lacking these functions. Neutrophils can reportedly kill tumor cells by Fc receptor-dependent cytotoxicity against antibody-opsonized cells or by secreting H_2_O_2_ to induce lethal Ca2+ influx (Furumaya et al., 2020). In our research, the expression levels of GINS1/4 negatively correlated with CD4+ T cell infiltration levels. Our results suggest that GINS subunits are potential targets and may affect the survival outcomes of sarcoma patients by interfering with immune infiltration. However, the specific molecular mechanism of GINS subunits on tumor immune regulation remains unclear, warranting further experiments.

In summary, our research comprehensively analyzed the expression and mutation of GINS subunits on immune infiltration and prognosis of SARC patients, providing further directions for studying the molecular biological properties of sarcoma. Our study suggested that all four members of GINS subunits were upregulated in sarcoma, and their up-regulation predicted shorter survival of SARC. Therefore, detecting the expression level of GINS genes in SARC tissue may be a promising strategy for predicting the prognosis of SARC patients. Meanwhile, the inhibition of GINS gene expression is possible to prolong sarcoma patients’ survival. Moreover, the potential ceRNA network was analyzed and constructed in this study. The construction of this network provides potential research directions for targeted inhibitors of GINS genes. In addition, we found correlations between GINS subunits expression and infiltration of various immune cells and found that CD4+ T cells and neutrophils may be favorable prognostic factors for SARC. Based on the above results, GINS subunits are expected to be potential prognostic markers and novel therapeutic targets for SARC. This study has several limitations, the sample size in our research was still small, and further experiments and clinical studies are needed to validate our findings.

## Data Availability

The datasets presented in this study can be found in online repositories. The names of the repository/repositories and accession number(s) can be found in the article/Supplementary Material.
